# Synthesis and biological evaluation of nojirimycin- and pyrrolidine-based trehalase inhibitors

**DOI:** 10.3762/bjoc.8.58

**Published:** 2012-04-05

**Authors:** Davide Bini, Francesca Cardona, Matilde Forcella, Camilla Parmeggiani, Paolo Parenti, Francesco Nicotra, Laura Cipolla

**Affiliations:** 1 Department of Biotechnology and Biosciences, University of Milano-Bicocca, Piazza della Scienza 2, 20126 Milano, Italy; 2 Department of Chemistry “Ugo Schiff”, University of Florence, Polo Scientifico e Tecnologico, Via della Lastruccia 3–13, 50019 Sesto Fiorentino, Florence, Italy; 3 CNR-INO U. O. S. Sesto Fiorentino c/o LENS, Via Nello Carrara 1, 50019 Sesto Fiorentino, Florence, Italy; 4 Department of Environmental Sciences, University of Milano-Bicocca, Piazza della Scienza 1, 20126 Milano, Italy

**Keywords:** glycosidases inhibitors, iminosugars, nojirimycins, pyrrolidines, trehalases

## Abstract

A small set of nojirimycin- and pyrrolidine-based iminosugar derivatives has been synthesized and evaluated as potential inhibitors of porcine and insect trehalases. Compounds **12**, **13** and **20** proved to be active against both insect and porcine trehalases with selectivity towards the insect glycosidase, while compounds **10**, **14** and **16** behaved as inhibitors only of insect trehalase. Despite the fact that the activity was found in the micromolar range, these findings may help in elucidating the structural features of this class of enzymes of different origin, which are still scarcely characterised.

## Introduction

**Figure 1:**
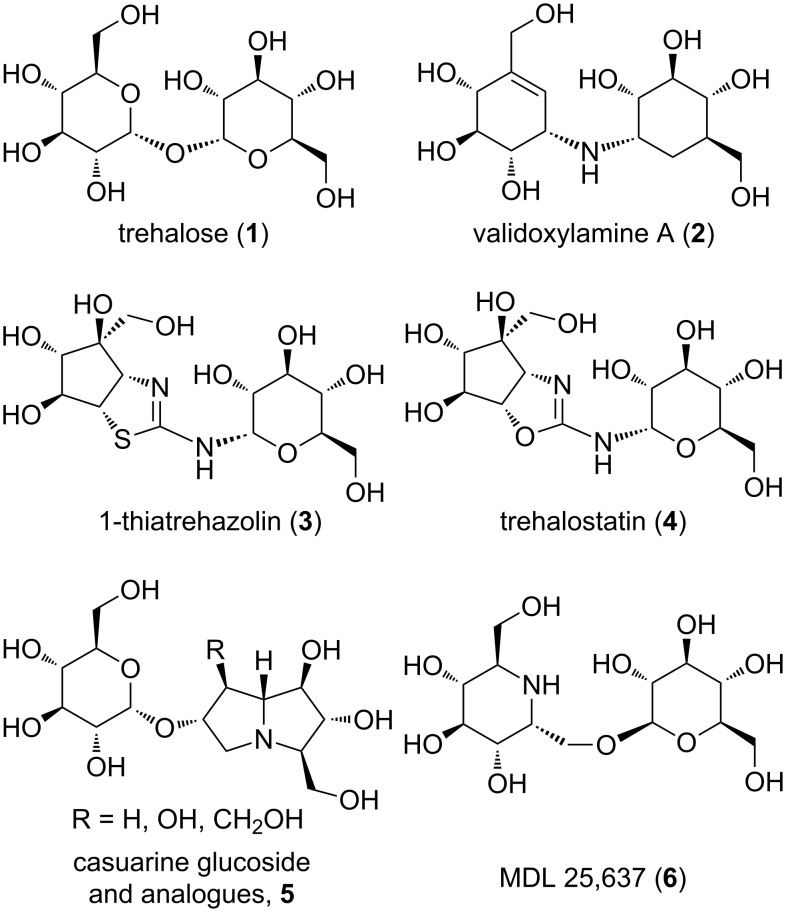
Structure of trehalose (**1**), validoxylamine A (**2**), 1-thiatrehazolin (**3**), trehalostatin (**4**), casuarine glucoside and analogues **5**, and MDL 25,637 (**6**).

Trehalase (EC3.2.1.28) is a glycosidase that catalyses trehalose (α-D-glucopyranosyl-α-D-glucopyranoside **1**, Figure 1) [1–3] hydrolysis. It was found initially at the end of the 19^th^ century in *Aspergillus niger* [4] and *S. cerevisiae* [5], and has since then been reported in several other organisms, including mammals, where it is found both in the kidney brush border membranes [6] and in the intestinal villae membranes [7]. While the role of trehalase in the kidney has not been elucidated yet (trehalose is absent in blood), in the intestine it hydrolyses ingested trehalose [8]. However, trehalose hydrolysis is fundamental for insect flight [9], growth resumption of resting cells, and spore germination in fungi.

Trehalase is an inverting glycosidase [10], belonging to the GH37 family of the carbohydrate-active enzyme (CAZy) classification [11], and despite its abundance in nature, few details are known of its function and properties. The first 3D structure of a trehalase (Tre37A from *E. coli*) in a complex with inhibitors (validoxylamine A (**2**) and 1-thiatrehazolin (**3**) Figure 1; protein data bank (PDB) entries 2JF4 and 2JG0 [12]) shows the presence of two subsites: Subsite +1 accommodating the leaving-group, the “recognition” site, and subsite −1 as the “catalytic” site.

Due to the biological relevance of trehalose and trehalase, several trehalose mimetics have been proposed as potential fungicides or antibiotics [13], such as trehalostatin (**4**) [1,14] and some iminosugar glycoconjugates, e.g., **5** or MDL 25,637 (**6**) [1,15–16] (Figure 1). In this work we report the synthesis and the biological activity of a small set of nojirimycin- and pyrrolidine-based iminosugar derivatives and their preliminary biological evaluation as inhibitors against porcine and insect trehalase from *C. riparius*.

## Results and Discussion

**Figure 2:**
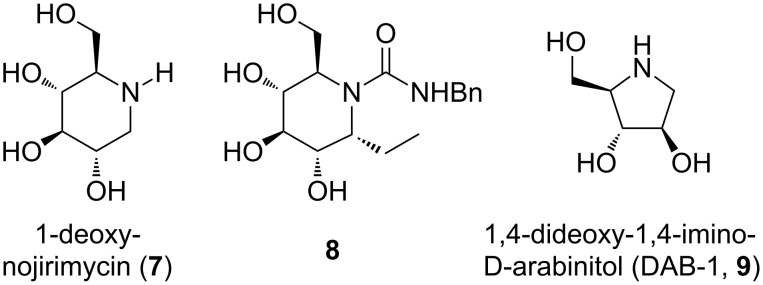
Structure of nojirimycin-based (**7**, **8**) and pyrrolidine-based (**9**) leads.

In previous studies by us and other research groups it was reported that 1-deoxynojirimycin (**7**) and its benzyl urea derivative **8** (Figure 2) [17–18] are trehalase inhibitors. It is worth noting that they have the nojirimycin ring in common with the trehalose mimetic compound **6** (Figure 1). Furthermore, it was also reported that pyrrolidine derivatives (i.e., DAB-1, **9**, Figure 2) [19] may act as trehalase inhibitors, in particular as competitive inhibitors with affinity to the catalytic site [19]. In general, it is well known that a key issue in the design of glycosidase inhibitors is specificity, for example, 1-deoxynojirimycin (**7**) is a glycosidase inhibitor in the low micromolar range, but despite its activity it lacks specificity. In this study we wish to gain further insights into the recognition requirements of the catalytic site of porcine (as the mammalian counterpart) and insect trehalase from *Chironomus riparius*. Both nojirimycin and pyrrolidine derivatives fall into the class of catalysis-site-targeting inhibitors [19].

**Figure 3:**
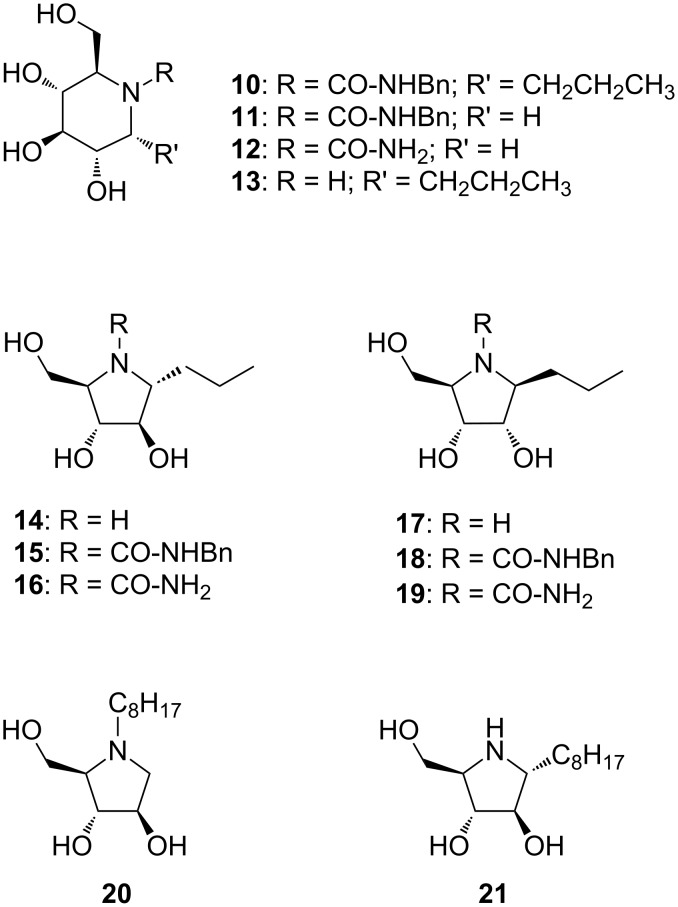
Structures of potential inhibitors **10**–**21**.

On the basis of these considerations, we designed and synthesized nojirimycin and pyrrolidine derivatives **10**–**21** (Figure 3), bearing different groups on the nitrogen atom and on the adjacent carbon. We did not expect a high value of inhibition, since, as already reported [19], good inhibitors must have a pseudodisaccharide structure, which ensures the synergistic interactions of an aminocyclitol or a nitrogen-containing heterocycle with the catalytic site, and of a sugar or cyclitol unit with the recognition site. However, this work may highlight relevant structural features of the catalytic site that can give access to specific inhibitors.

In general, the compounds were synthesized with the aim of understanding whether the presence of substituents on the nitrogen atom and/or a short- or medium-sized alkyl chain at position 1 (numbering of the parent aldose) can somehow influence the activity and selectivity. In addition, the pyrrolidine derivatives **14**–**16** and **17–19** possess a “α-D-arabino” and a “β-D-ribo” configuration, respectively, which may affect the activity and selectivity towards porcine and insect trehalase. Finally, we also included two pyrrolidine derivatives **20** and **21**, differing in the alkylation position with a C_8_ alkyl chain (Figure 3). These two compounds can help answer whether a medium-sized lipophylic chain can be accommodated into the catalytic site, and whether any difference could be due to the positioning of the chain itself. Only the “α-D-arabino-configured” pyrrolidines **20**, **21** were considered here, since preliminary data showed that “β-D-ribo-configured” pyrrolidines were not active at all (for details, see Enzyme assays).

### Chemical synthesis

Based on the structure of lead compound **8** (Figure 2), which showed some selectivity towards insect trehalase from *C.riparius* [18], we envisaged the possibility to synthesize a few nojirimycin and pyrrolidine derivatives bearing a benzyl urea moiety and a different alkyl substituent on the adjacent carbon (**10**, **11**, **15** and **18**, Figure 3). Thus the presence of a benzyl urea moiety was expected to be a common feature of the majority of the iminosugar derivatives (piperidines and pyrrolidines). However, during the final deprotection step by hydrogenolysis, the reaction resulted in the formation of the disubstituted urea **10** or, unexpectedly, monosubstituted ureas **12**, **16** and **19** (Figure 3), depending on the starting material.

Pyrrolidine derivatives were synthesized with different stereochemistry on the five-membered ring (i.e., compounds **14**, **16** versus **17**, **19**, Figure 3), in order to elucidate whether this feature could be relevant for enzyme recognition, and with a sterically demanding alkyl chain positioned either at the nitrogen atom or at the adjacent carbon (i.e., compounds **20** and **21**, Figure 3).

#### Nojirimycin-based derivatives **10**, **12** and **13**

**Scheme 1:**
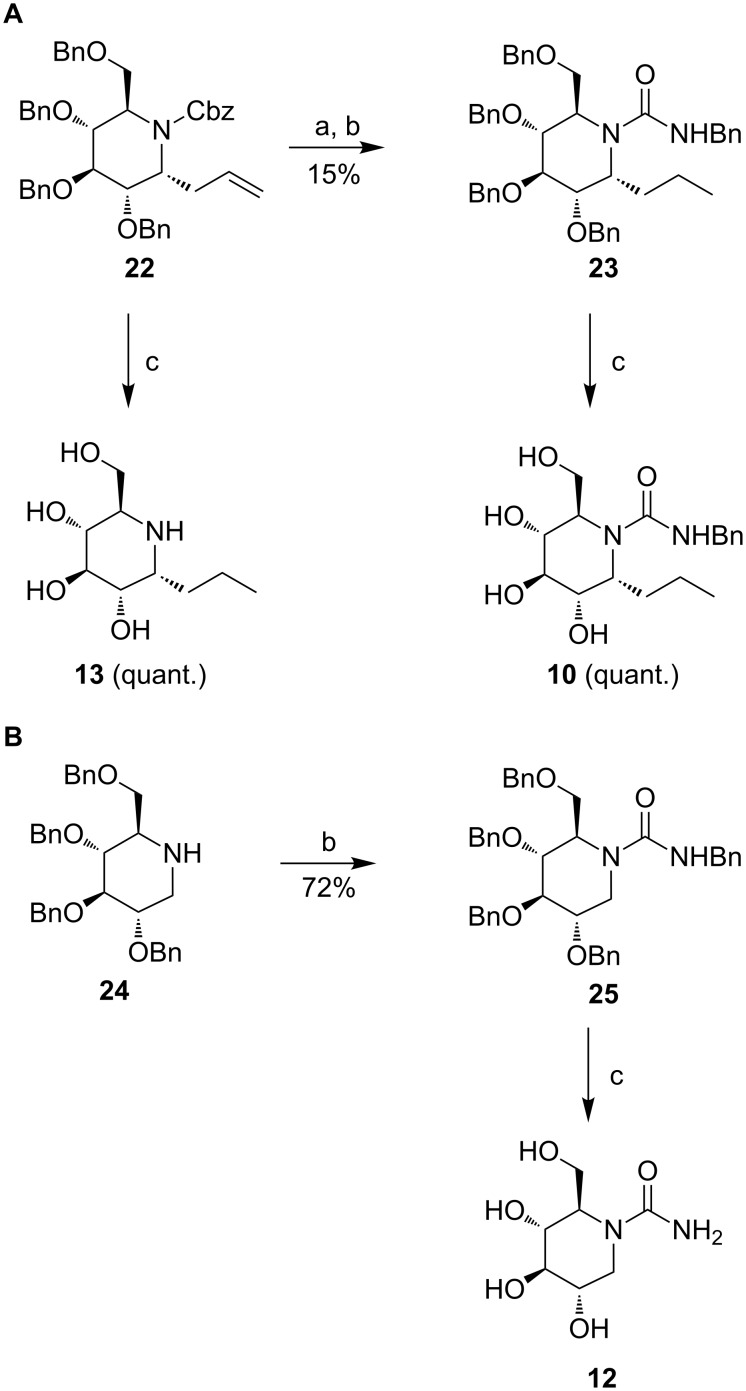
Synthesis of nojirimycin-based inhibitors **10**,**12** and **13**. Reagents and conditions: (a) H_2_, Pd/C, NH_4_OAc, EtOH, rt, 10 h; (b) benzyl isocyanate, DME, 85 °C, 2 h; (c) H_2_, Pd(OH)_2_/C, EtOAc/EtOH 1:1, rt, 5 d.

Compounds **10** and **12** were synthesized from the corresponding protected nojirimycin derivatives **22** [20] (Scheme 1A) and **24** [21] (Scheme 1B). Cbz deprotection of compound **22** (Scheme 1A) followed by reaction with benzyl isocyanate in dimethoxyethane at 85 °C afforded urea **23** (15% yield over two steps). Reaction of compound **24** directly with benzyl isocyanate in dimethoxyethane at 85 °C afforded urea **25** in 72% yield (Scheme 1B).

The hydrogenolysis of benzyl ureas **23** and **25** unexpectedly proceeded in a different manner. Derivative **23** afforded benzyl nojirimycin urea **10** in quantitative yield (Scheme 1A), while derivative **25**, under the same reaction conditions gave monosubstituted urea **12** in 83% purity, as determined by NMR (Scheme 1B). Impurities, which could not be separated from the title compound, were due to small amounts of the benzyl urea that could not be fully hydrolysed, even after prolonged reaction times.

In order to figure out whether the benzyl urea moiety might have any effect on the activity and specificity against trehalases, derivative **13** was also synthesized by direct hydrogenolysis of starting compound **22** (Scheme 1A). Any activity difference between inhibitor **10** and **13** must be ascribed to the presence of the benzyl urea group instead of the free nojirimycin NH.

#### Pyrrolidine-based compounds **14**, **16**, **17** and **19**–**21**

**Scheme 2:**
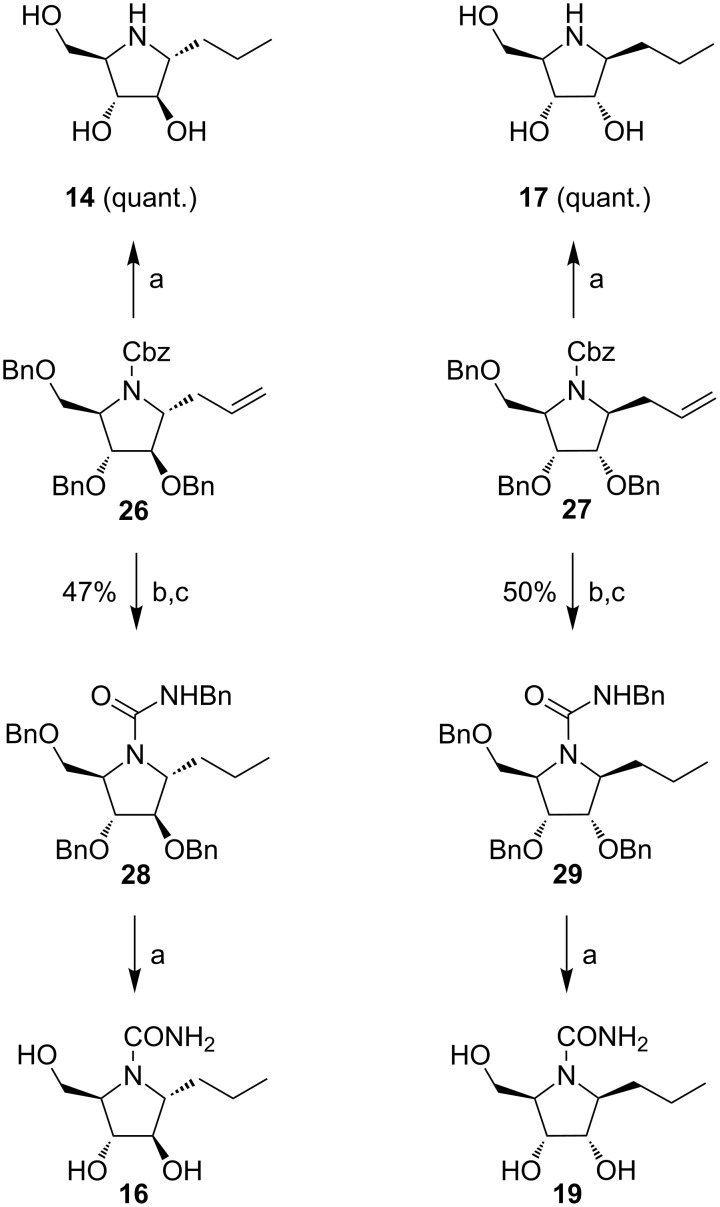
Synthesis of pyrrolidine derivatives **14**, **16**, **17** and **19**. Reagents and conditions: (a) H_2_, Pd(OH)_2_/C, EtOAc/EtOH 1:1, rt, 5 d; (b) H_2_, Pd/C, NH_4_OAc, EtOH, rt, 10 h; (c) benzyl isocyanate, DME, 85 °C, 2 h.

Pyrrolidine derivatives **14**, **16**, **17** and **19** were obtained from the corresponding pyrrolidines **26** and **27** [22], by following the same synthetic steps used for nojirimycin derivatives, as outlined in Scheme 2. Direct hydrogenolysis of **26** and **27** afforded quantitatively the compounds **14** and **17**, respectively. Cbz deprotection of **26** and **27** followed by reaction with benzyl isocyanate in dimethoxyethane at 85 °C produced ureas **28** and **29** in 47 and 50% overall yields, respectively. As previously observed, hydrogenolysis of **28** and **29** afforded monosubstituted ureas **16** and **19**, with loss of the *N*-benzyl group. In addition, while derivative **19** was obtained with comparable purity (85%) to compound **12**, deprotection of intermediate **28** afforded monosubstituted urea **16** in only 58% purity.

**Scheme 3:**
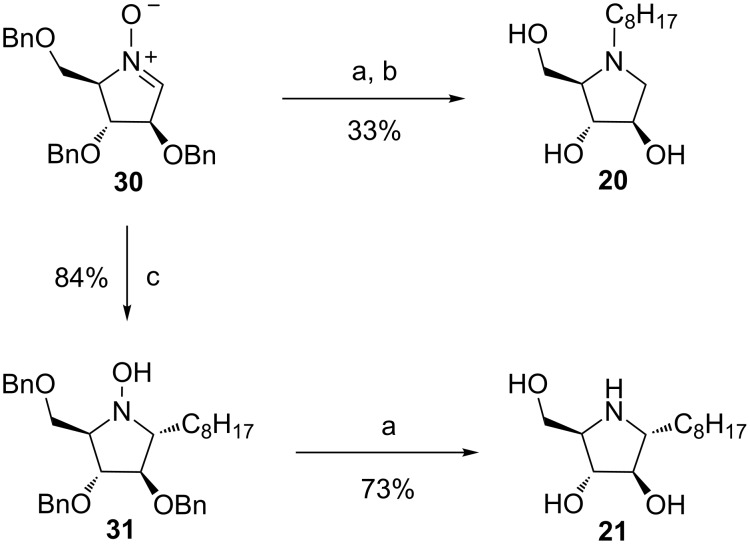
Synthesis of pyrrolidines **20** and **21**. Reagents and conditions: (a) H_2_, Pd/C, MeOH, HCl; (b) octanal, NaBH_3_CN, MeOH, AcOH rt; (c) C_8_H_17_MgBr (2 M in Et_2_O), THF, −75 °C to rt (3 h).

In addition, pyrrolidines **20** and **21** were synthesized in a few steps from nitrone **30** [23]. Catalytic hydrogenation over Pd/C followed by reductive amination in the presence of octanal and NaBH_3_CN afforded compound **20** in 33% yield over two steps (Scheme 3). Grignard addition of octylmagnesium bromide to nitrone **30** proceeded cleanly and gave stereoselectively the “all trans” hydroxypyrrolidine **31** as a single adduct in 84% yield, with a stereoselectivity that was in accordance with previously reported Grignard additions on the same nitrone [24]. Final catalytic hydrogenation over Pd/C gave pyrrolidine **21**, which was recently synthesized by an enantioselective strategy [25], in 73% yield.

### Enzyme assays

**Figure 4:**
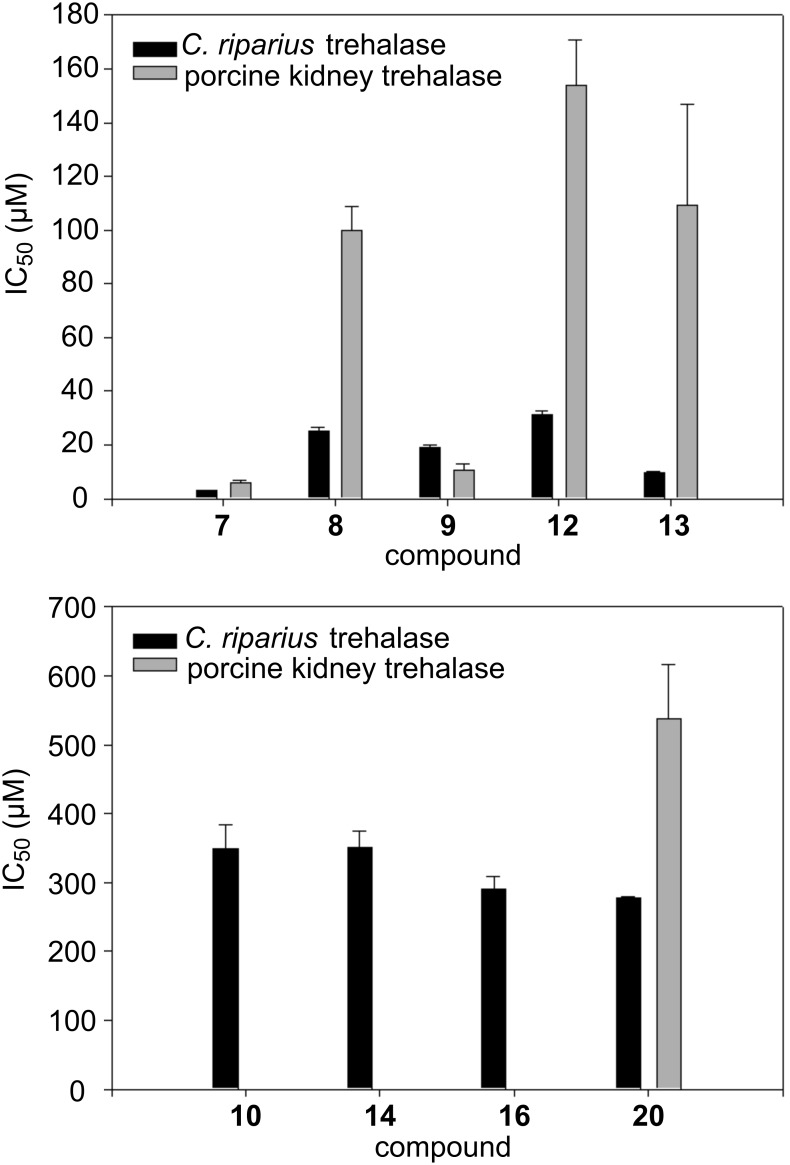
Histogram of the inhibitory activity of compounds **7**–**10**, **12**–**14**, **16** and **20**. Derivatives **10**, **14** and **16** showed no activity against porcine kidney trehalase.

Synthesized compounds **10**, **12**–**14**, **16**, **17** and **19–21** were tested for their inhibitory activity against insect (*C. riparius*) and porcine kidney trehalase. All data are summarised in Figure 4 and Table 1.

**Table 1:** Inhibition of trehalase activity. Fixed amounts of *C. riparius* and kidney porcine trehalases were incubated in the presence of fixed concentrations (*K*_m_) of trehalose and increasing concentrations of the indicated inhibitors. Parameters were calculated as described in the text. Data are (means ± SE) of three independent experiments.

Compound	IC_50_ *C. riparius* trehalase (µM)	IC_50_ porcine kidney trehalase (µM)

**7**	2.8 ± 0.34^a^	5.96 ± 0.62^b^
**8**	25.0 ± 1.60^a^	100.0 ± 8.82^a^
**9**	19.0 ± 0.95^a^	10.6 ± 2.42^a^
**10**	349.0 ± 35	no inhibition
**12**	31.0 ± 1.82	154.0 ± 17
**13**	9.70 ± 0.30	109.0 ± 38
**14**	350.0 ± 24	no inhibition
**16**	290.0 ± 18	no inhibition
**17**	no inhibition	no inhibition
**19**	no inhibition	no inhibition
**20**	277.0 ± 2.63	537.0 ± 80
**21**	no inhibition	no inhibition

^a^values from [18]; ^b^values from [13].

Even if the synthesis unexpectedly afforded a structurally quite heterogeneous set of compounds, biological data give some hints toward the design of selective inhibitors of trehalases of different origin. Trehalase activity was measured through a coupled assay with glucose-6-phosphate dehydrogenase and hexokinase according to Wegener et al. [26]. To examine the potential of each compound as a trehalase inhibitor, screening assays of potential inhibitors were carried out at a fixed concentration of 1 mM. For the most active compounds, dose–response curves were established to determine the IC_50_ values. Experiments were performed in the presence of increasing concentrations of the inhibitor at a fixed substrate concentration close to the *K*_m_ value (0.5 mM for *C. riparius* trehalase and 2.5 mM for porcine trehalase). Initial rates as a function of inhibitor concentration were fitted to the following equation:


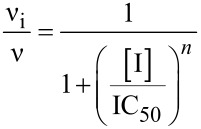


where ν_i_ and ν are the initial rate in the presence and in the absence of the inhibitor, respectively, [I] is the inhibitor concentration, IC_50_ is the inhibitor concentration producing half-maximal inhibition, and *n* is the Hill coefficient.

In the nojirimycin series (compounds **10**, **12** and **13**) the most active compound was derivative **13**, with IC_50_ values close to 1-deoxynojirimycin (**7**) for insect trehalase inhibition (Table 1). Interestingly, compound **13** was found to be around ten times more active towards insect trehalase than to porcine trehalase, hence more specific than lead compound **7** (only twice as active on insect trehalase), suggesting that a short alkyl chain at C-1, together with the free NH group in the ring account for the good activity and specificity.

Comparing the activity of lead **7** with compound **12**, it appears that substitution on the nitrogen atom of the ring causes a drop in activity; however, a good degree of selectivity (five times) towards the insect glycosidase is maintained.

When substituents are introduced both on the nitrogen of the ring and at C-1, as in compound **10**, a further drop of activity can be observed for insect trehalase inhibition (IC_50_ 2.8 μM → 31.0 μM → 349.0 μM for **7** → **12** → **10**), together with complete loss of activity against the porcine enzyme (IC_50_ 5.96 μM → 154.0 μM → no inhibition for **7** → **12** → **10**), thus maximising selectivity. The inhibition of the trehalase activity of **8** is 25 μM and of **10** is 349 μM: Elongation of the ethyl chain of compound **8** to the propyl group of inhibitor **10** causes a more than ten-fold drop in activity against insect trehalase.

Analysis of the pyrrolidine set (**14**, **16**, **17** and **19**–**21**) immediately shows that the “β-D-ribo-configured” pyrrolidines **17** and **19** do not possess any activity against either enzyme. As for the “α-D-arabino-configured” pyrrolidines, the presence of a sterically demanding substituent (C_8_ alkyl chain) at C-1 (compound **21**) is detrimental for the inhibition of both trehalases, while shorter chains, as in **14**, are accepted only by the catalytic site of insect trehalase, thus imparting selectivity in inhibition. In contrast, when the C_8_ alkyl chain is positioned on the nitrogen (compound **20**) both enzymes can accommodate the inhibitor in the catalytic pocket, with a preference for the insect trehalase. Furthermore, the presence of substituents both on the nitrogen of the ring and at C-1, as in compound **16**, slightly increases the activity against insect trehalase (IC_50_ 350.0 μM → 290.0 μM for **14** → **16**).

It is worth noting that the introduction of small substituents on lead pyrrolidine **9**, either at the nitrogen atom or at the adjacent carbon, affords compounds less active than **9**, but with reversed specificity (**9** is twice more active on porcine trehalase, while **14**, **16** and **20** are more active on insect enzyme).

## Conclusion

The design and synthesis of enzyme inhibitors can often provide information about the mechanism of action and chemical topography of the active site of the enzyme under consideration. We proposed the synthesis of a small set of iminosugar derivatives, which in some cases resulted in selective inhibitors of trehalases of different origin, despite the fact that their activity was in the micromolar range. The most active and specific inhibitor was compound **13**, characterised by a nojirimycin ring with a propyl group at C-1. Compared to lead 1-deoxynojirimycin (**7**), the presence of the propyl group in **13** causes a slight decrease of activity, but nevertheless imparting a ten-fold selectivity towards insect trehalase. In general, the collected data clearly indicate that the catalytic sites of trehalases from porcine kidney and insects have different recognition requirements, which can be exploited for the future design of specific inhibitors.

Further studies are needed in order to characterise the synthesized compounds in terms of their inhibitory activity against other glycosidases of interest, such as maltase, isomaltase, sucrase, glucoamylase, lactase and α-amylase.

## Experimental

### Synthesis

#### General methods

Solvents were dried over molecular sieves for at least 24 h prior to use, when required. When dry conditions were required, the reaction was performed under Ar or N_2_ atmosphere. Thin-layer chromatography (TLC) was performed on silica gel 60F_254_ coated glass plates (Merck) with UV detection when possible, or spots were visualized by charring with a conc. H_2_SO_4_/EtOH/H_2_O solution (10:45:45 v/v/v), or with a solution of (NH_4_)_6_Mo_7_O_24_ (21 g), Ce(SO_4_)_2_ (1 g), conc. H_2_SO_4_ (31 mL) in water (500 mL) and then heating to 110 °C for 5 min. Flash column chromatography was performed on silica gel 230–400 mesh (Merck). Routine ^1^H and ^13^C NMR spectra were recorded on a Varian Mercury instrument at 400 MHz (^1^H) and 100.57 MHz (^13^C) or on a Varian Gemini 200 MHz instrument 50.29 MHz (^13^C) where stated. Chemical shifts are reported in parts per million downfield from TMS as an internal standard; *J* values are given in Hz. Mass spectra were recorded on a System Applied Biosystems MDS SCIEX instrument (Q TRAP, LC/MS/MS, turbo ion spray) or on a System Applied Biosystem MDS SCIEX instrument (Q STAR elite nanospray). ESI full MS were recorded on a Thermo LCQ instrument by direct inlet; relative percentages are shown in brackets. Elemental analyses (C, H, N) were performed on a Perkin-Elmer series II 2400 analyzer, and all synthesized compounds showed a purity of more than 95%.

**General procedure for hydrogenolysis (compounds 10**, **12**, **13**, **14**, **16**, **17**, **19):** A 0.02 M solution of the appropriate compound dissolved in EtOAc/EtOH 1:1 was treated with Pd(OH)_2_/C (100 wt %). The reaction mixture was stirred for 5 d under a H_2_ atmosphere. Palladium was then removed by filtration through a Celite pad followed by washing with EtOH and water. Evaporation of the solvents afforded the corresponding deprotected compounds in quantitative yields.

**General procedure for Cbz deprotection:** To a 0.2 M solution of the appropriate compound dissolved in EtOH, crystallized NH_4_OAc (0.5 equiv) and Pd/C (5 wt %) were added. The reaction mixture was stirred overnight under a H_2_ atmosphere. Palladium was then removed by filtration through a Celite pad followed by washing with EtOH. The solvent was removed under reduced pressure and crude amine was used for the benzyl isocyanate reaction (see general procedure for details).

**General procedure for benzyl isocyanate reaction:** To a 0.07 M solution of the appropriate compound dissolved in dry DME, benzyl isocyanate (2 equiv) was added and the reaction mixture was heated under reflux. After 2 h the solvent was evaporated under reduced pressure. The residue was purified on a silica gel column with a suitable eluent. See Supporting Information File 1 for full experimental data.

#### Enzyme assays

All enzyme assays were performed in triplicate at 30 °C by using sample volumes varying from 5 to 20 µL in 1 mL test tubes and using a Cary3 UV–vis spectrophotometer. Enzyme activities were analyzed by Cary Win UV application software for Windows XP. The specific activity (U mg^−1^) was expressed as µmol min^−1^(mg protein)^−1^. Values were expressed as mean ± SE of replicated.

## Supporting Information

File 1 Full experimental data.
