# Supplementary material for: Synthesis and biological evaluation of nojirimycin- and pyrrolidine-based trehalase inhibitors
Source: Beilstein J Org Chem. 2012 Apr 5;8:514–21. doi: 10.3762/bjoc.8.58 (PMC3326631; doi:10.3762/bjoc.8.58)
Supplement: File 1 — Full experimental data. [file Beilstein_J_Org_Chem-08-514-s001.pdf]

Supporting Information

for

Synthesis and biological evaluation of nojirimycin- and pyrrolidine-based trehalase inhibitors

Davide Bini[1], Francesca Cardona[2], Matilde Forcella[1], Camilla Parmeggiani[2,3], Paolo Parenti[4], Francesco Nicotra[1], Laura Cipolla*[1]

Address: [1]Department of Biotechnology and Biosciences, University of Milano-Bicocca, Piazza della Scienza 2, 20126 Milano, Italy, [2]Department of Chemistry "Ugo Schiff", University of Florence, Polo Scientifico e Tecnologico, Via della Lastruccia 3–13, 50019 Sesto Fiorentino, Florence, Italy, [3]Present address: CNR-INO U. O. S. Sesto Fiorentino c/o LENS, Via Nello Carrara 1, 50019 Sesto Fiorentino, Florence, Italy and [4]Department of Environmental Sciences, University of Milano-Bicocca, Piazza della Scienza 1, 20126 Milano, Italy.

Email: Laura Cipolla - laura.cipolla@unimib.it

* Corresponding author

Full experimental data

(2*R*,3*R*,4*R*,5*S*,6*R*)-*N*-benzyl-3,4,5-tris(benzyloxy)-2-((benzyloxy)methyl)-6-propylpiperidine-1-carboxamide (23): Flash column chromatography (petroleum ether/EtOAc, 87.5:12.5). ^{1}H NMR (400 MHz, $CDCl_3$) δ 7.38–7.00 (m, 25H, ArH), 6.75 (bs, 1H, NH), 4.87–4.20 (m, 10H, CH_2Ph), 4.02–3.58 (m, 7H, H-2, H-3, H-4, H-5, H-6, H-1’), 1.78–1.32 (m, 4H, $CH_2CH_2CH_3$), 0.90 (t, *J* = 7.3 Hz, 3H, $CH_2CH_2CH_3$) ppm; Anal. calcd for $C_{45}H_{50}N_2O_5$: C, 77.33; H, 7.21; N, 4.01; found: C, 77.30; H, 7.19; N, 3.99.

(2*R*,3*R*,4*R*,5*S*,6*R*)-*N*-benzyl-3,4,5-trihydroxy-2-(hydroxymethyl)-6-propylpiperidine-1-carboxamide (10): ^{1}H NMR (400 MHz, D_2O) δ 7.36–7.13 (m, 5H, ArH), 4.82–4.54 (m, 2H, H-1’), 4.44 (d, *J* = 15.2 Hz, 1H, CH_2Ph), 4.33 (d, *J* = 15.1 Hz, 1H, CH_2Ph), 4.06–3.98 (m, 1H, H-2), 3.97–3.89 (m, 1H, H-6), 3.66–3.34 (m, 3H, H-3, H-4, H-5), 1.72–1.61 (m, 1H, $CH_2CH_2CH_3$), 1.52–1.38 (m, 1H, $CH_2CH_2CH_3$), 1.28–1.18 (m, 1H, $CH_2CH_2CH_3$), 1.14–1.04 (m, 1H, $CH_2CH_2CH_3$), 0.90–0.72 (m, 3H, $CH_2CH_2CH_3$) ppm; Anal. calcd for $C_{17}H_{26}N_2O_5$: C, 60.34; H, 7.74; N, 8.28; found: C, 60.31; H, 7.76; N, 8.25.

(2*R*,3*R*,4*R*,5*S*,6*R*)-2-(hydroxymethyl)-6-propylpiperidine-3,4,5-triol (13): ^{1}H NMR (400 MHz, D_2O) δ 3.69–3.63 (m, 1H, H-4), 3.60–3.40 (m, 5H, H-3, H-5, H-6, H-1’), 3.23–3.13 (m, 1H, H-2), 1.51–1.36 (m, 2H, $CH_2CH_2CH_3$), 1.35–1.19 (m, 2H, $CH_2CH_2CH_3$), 0.77 (t, *J* = 7.3 Hz, 3H, $CH_2CH_2CH_3$) ppm; ^{13}C NMR (100.57 MHz, D_2O) δ 71.85 (d, C-5), 68.94 (d, C-4), 67.79 (d, C-3), 62.46 (t, C-1’), 55.32 (d, C-2), 55.21(d, C-6), 26.30 (t, $CH_2CH_2CH_3$), 19.15 (t, $CH_2CH_2CH_3$), 12.87 (q, $CH_2CH_2CH_3$) ppm; MS (TOF) *m/z*: 206.1 $[M + H]^+$; found 206.3; Anal. calcd for $C_9H_{19}NO_4$: C, 52.66; H, 9.33; N, 6.82; found: C, 52.68; H, 9.31; N, 6.80.

(2*R*,3*R*,4*R*,5*S*)-*N*-benzyl-3,4,5-tris(benzyloxy)-2-((benzyloxy)methyl)piperidine-1-carboxamide (25): Flash column chromatography (petroleum ether/EtOAc 75:25).
^{1}H NMR (400 MHz, $CDCl_3$) δ 7.32–7.17 (m, 25H, ArH), 5.85 (bs, 1H,NH), 4.73 (dd, *J* = 11.6, 8.1 Hz, 2H, CH_2Ph), 4.63 (s, 2H, CH_2Ph), 4.52 (dd, *J* = 21.0, 11.6 Hz, 2H, CH_2Ph), 4.40 (d, *J* = 2.7 Hz, 2H, CH_2Ph), 4.32 (d, *J* = 3.5 Hz, 2H, NHCH_2Ph), 4.09–4.03 (m, 1H, H-

2), 3.96 (dd, J = 14.1, 4.4 Hz, 1H, Ha-6), 3.80–3.73 (m, 1H, H-4), 3.70 (dd, J = 8.8, 4.4 Hz, 1H, H-3), 3.63 (dd, J = 9.6, 3.5 Hz, 1H, Ha-1'), 3.60–3.53 (m, 2H, H-5, Hb-1'), 3.32 (dd, J = 14.1, 3.7 Hz, 1H, Hb-6) ppm; ^{13}C NMR (100.57 MHz, $CDCl_3$) δ 159.45 (s, C=O), 139.82, 138.36, 138.24, 138.12, 137.67 (s, CAr), 128.68–127.16 (d, CHAr), 82.33 (d, C-4), 79.05 (d, C-3), 76.04 (d, C-5), 73.60, 73.24 (t, CH_2Ph), 71.22 (t, C-1'), 57.31 (d, C-2), 45.20 (t, NH*C*H_2Ph), 41.00 (t, C-6) ppm; Anal. calcd for $C_{42}H_{44}N_2O_5$: C, 76.80; H, 6.75; N, 4.26; found: C, 76.78; H, 6.71; N, 4.28.

(2*R*,3*R*,4*R*,5*S*)-3,4,5-trihydroxy-2-(hydroxymethyl)piperidine-1-carboxamide (12): ^{1}H NMR (400 MHz, D_2O) δ 3.74–3.69 (m, 1H, H-2), 3.69–3.54 (m, 4H, H-5, Ha-6, H-1'), 3.54–3.49 (m, 1H, H-3), 3.45–3.40 (m, 1H, H-4), 3.22 (dd, J = 14.9, 3.2 Hz, 1H, Hb-6) ppm; ^{13}C NMR (100.57 MHz, D_2O) δ 161.15 (s, C=O), 73.11 (d, C-4), 70.59 (d, C-5), 68.07 (d, C-3), 59.72 (d, C-2), 59.46 (t, C-1'), 43.78 (t, C-6) ppm; MS (TOF) *m/z*: 207.1 $[M + H]^+$; found 207.3.

(2*R*,3*R*,4*R*,5*R*)-2-(hydroxymethyl)-5-propylpyrrolidine-3,4-diol (14): ^{1}H NMR (400 MHz, D_2O) δ 3.89 (dd, J = 13.7, 6.5 Hz, 1H, H-3), 3.83–3.64 (m, 3H, H-4, H-1'), 3.39 (bs, 1H, H-2), 3.26 (dd, J = 14.1, 8.0 Hz, 1H, H-5), 1.70 (dt, J = 15.1, 6.4 Hz, 1H, $CH_2CH_2CH_3$), 1.57 (dt, J = 14.2, 8.7 Hz, 1H, $CH_2CH_2CH_3$), 1.28 (dt, J = 14.7, 7.5 Hz, 2H, $CH_2CH_2CH_3$), 0.78 (t, J = 7.1 Hz, 3H, $CH_2CH_2CH_3$) ppm; ^{13}C NMR (100.57 MHz, D_2O) δ 77.98 (d, C-4), 74.06 (d, C-3), 61.92 (d, C-2), 61.09 (d, C-5), 57.78 (t, C-1'), 32.08 (t, $CH_2CH_2CH_3$), 18.58 (t, $CH_2CH_2CH_3$), 12.73 (q, $CH_2CH_2CH_3$) ppm; MS (TOF) *m/z*: 176.1 $[M + H]^+$; found 176.2; Anal. calcd for $C_8H_{17}NO_3$: C, 54.83; H, 9.78; N, 7.99; found: C, 54.80; H, 10.00; N, 8.01.

(2*R*,3*R*,4*S*,5*S*)-2-(hydroxymethyl)-5-propylpyrrolidine-3,4-diol (17): ^{1}H NMR (400 MHz, D_2O) δ 4.10 (t, J = 5.4 Hz, 1H, H-3), 3.97 (t, J = 5.8 Hz, 1H, H-4), 3.82 (dd, J = 12.6, 3.8 Hz, 1H, Ha-1'), 3.73 (dd, J = 12.6, 6.0 Hz, 1H, Hb-1'), 3.55 (dd, J = 9.4, 4.6 Hz, 1H, H-2), 3.45 (dd, J = 14.1, 7.2 Hz, 1H, H-5), 1.76–1.56 (m, 2H, $CH_2CH_2CH_3$), 1.43–1.29 (m, 2H, $CH_2CH_2CH_3$), 0.85 (t, J = 7.3 Hz, 3H, $CH_2CH_2CH_3$) ppm; ^{13}C NMR (100.57 MHz, D_2O) δ

74.26 (d, C-4), 71.09 (d, C-3), 64.97 (d, C-2), 63.60 (d, C-5), 59.13 (t, C-1'), 32.50 (t, $\mathit{C}H_2CH_2CH_3$), 19.86 (t, $CH_2\mathit{C}H_2CH_3$), 13.69 (q, $CH_2CH_2\mathit{C}H_3$) ppm; MS (TOF) *m/z*: 176.1 $[M + H]^+$; found 176.2; Anal. calcd for $C_8H_{17}NO_3$: C, 54.83; H, 9.78; N, 7.99; found: C, 54.85; H, 9.75; N, 7.96.

(2*R*,3*R*,4*R*,5*R*)-*N*-benzyl-3,4-bis(benzyloxy)-2-((benzyloxy)methyl)-5-propylpyrrolidine-1-carboxamide (28): Flash column chromatography (petroleum ether/EtOAc 75:25). ^{1}H NMR (400 MHz, $CDCl_3$) δ 7.41–7.10 (m, 20H, ArH), 5.50 (bs, 1H, NH), 4.64–4.30 (m, 8H, CH_2Ph), 4.14–4.05 (m, 1H, H-2), 3.99 (m, 1H, H-4), 3.91 (m, 1H, H-5), 3.85–3.69 (m, 2H, H-3, Ha-1'), 3.58–3.50 (m, 1H, Hb-1'), 2.03 (m, 1H, $C\mathit{H_2}CH_2CH_3$), 1.61 (m, 1H, $C\mathit{H_2}CH_2CH_3$), 1.37–1.20 (m, 2H, $CH_2C\mathit{H_2}CH_3$), 0.91 (t, J = 7.3 Hz, 3H, $CH_2CH_2C\mathit{H_3}$) ppm; ^{13}C NMR (100.57 MHz, $CDCl_3$) δ 157.67 (s, C=O), 140.57–138.56 (s, C Ar), 129.38–127.95 (d, CH Ar), 83.89 (d, C-4), 82.90 (d, C-3), 74.10–71.73 (t, CH_2Ph, C-1'), 65.42 (d, C-5), 63.77 (d, C-2), 45.54 (t, $NH\mathit{C}H_2Ph$), 33.55 (t, $\mathit{C}H_2CH_2CH_3$), 20.89 (t, $CH_2\mathit{C}H_2CH_3$), 14.85 (q, $CH_2CH_2\mathit{C}H_3$) ppm; MS (TOF) *m/z*: 579.3 $[M + H]^+$; found 579.6; Anal. calcd for $C_{37}H_{42}N_2O_4$: C, 76.79; H, 7.31; N, 4.84; found: C, 76.81; H, 7.28; N, 4.81.

(2*R*,3*R*,4*S*,5*S*)-*N*-benzyl-3,4-bis(benzyloxy)-2-((benzyloxy)methyl)-5-propylpyrrolidine-1-carboxamide (29): Flash column chromatography (petroleum ether/EtOAc 25:75). ^{1}H NMR (400 MHz, $CDCl_3$) δ 7.41–7.11 (m, 20H, ArH), 6.73 (bs, 1H, NH), 4.75–4.23 (m, 9H, H-1, CH_2Ph), 4.05 (m, 1H, H-2), 3.76–3.66 (m, 3H, H-4, H-3, Ha-1'), 3.40 (t, J = 9.0 Hz, 1H, Hb-1'), 1.49–1.40 (m, 1H, $C\mathit{H_2}CH_2CH_3$), 1.39–1.31 (m, 1H, $C\mathit{H_2}CH_2CH_3$), 1.29–1.18 (m, 2H, $CH_2C\mathit{H_2}CH_3$), 0.90 (t, J = 7.2 Hz, 3H, $CH_2CH_2C\mathit{H_3}$) ppm; ^{13}C NMR (100.57 MHz, $CDCl_3$) δ 160.26 (s, C=O), 140.71, 138.74, 138.33, 137.85 (s, C Ar), 129.38–127.68 (d, CH Ar), 80.82 (d, C-4), 77.43 (d, C-3), 75.53, 74.21, 72.60, 71.43 (t, C-1', CH_2Ph), 61.94 (d, C-5, C-2), 45.48 (t, $NHCH_2Ph$), 37.35 (t, $\mathit{C}H_2CH_2CH_3$), 20.48 (t, $CH_2\mathit{C}H_2CH_3$), 15.02 (q, $CH_2CH_2\mathit{C}H_3$) ppm; MS (TOF) *m/z*: 579.3 $[M + H]^+$; found 579.6; Anal. calcd for $C_{37}H_{42}N_2O_4$: C, 76.79; H, 7.31; N, 4.84; found: C, 76.77; H, 7.33; N, 4.85.

(2*R*,3*R*,4*R*,5*R*)-3,4-dihydroxy-2-(hydroxymethyl)-5-propylpyrrolidine-1-carboxamide (16): ^{1}H NMR (400 MHz, D_2O) δ 3.95 (d, *J* = 12.8 Hz, 1H, H-3), 3.87 (d, *J* = 11.7 Hz, 1H, H-4), 3.65–3.54 (m, 3H, H-2, H-1'), 3.47–3.39 (m, 1H, H-5), 1.69–1.49 (m, 1H, $CH_2CH_2CH_3$), 1.47–1.31 (m, 1H, $CH_2CH_2CH_3$), 1.23–1.04 (m, 2H, $CH_2CH_2CH_3$), 0.73–0.67 (m, 3H, $CH_2CH_2CH_3$) ppm; ^{13}C NMR (100.57 MHz, D_2O) δ 158.15 (s, C=O), 77.74, 77.62 (d, C-3, C-4), 66.47 (d, C-2), 65.93 (d, C-5), 59.87 (t, C-1'), 31.43 (t, $CH_2CH_2CH_3$), 18.60 (t, $CH_2CH_2CH_3$), 13.05 (q, $CH_2CH_2CH_3$) ppm; MS (TOF) *m/z*: 219.1 $[M + H]^+$; found 219.2.

(2*R*,3*R*,4*S*,5*S*)-3,4-dihydroxy-2-(hydroxymethyl)-5-propylpyrrolidine-1-carboxamide (19): ^{1}H NMR (400 MHz, acetone) δ 4.30–4.04 (m, 1H, Ha-1'), 3.91 (bs, 1H, Hb-1'), 3.84 (d, *J* = 9.4 Hz, 1H, H-4), 3.81–3.75 (m, 1H, H-5), 3.70 (t, *J* = 6.0 Hz, 1H, H-2), 3.54–3.46 (m, 1H, H-3), 1.66–1.31 (m, 4H, $CH_2CH_2CH_3$), 0.91 (t, *J* = 7.0 Hz, 3H, $CH_2CH_2CH_3$) ppm; ^{13}C NMR (100.57 MHz, D_2O) δ 160.68 (s, C=O), 73.38, 71.94 (d, C-3, C-4), 63.97, 63.62 (d, C-2, C-5), 61.33 (t, C-1'), 34.18 (t, $CH_2CH_2CH_3$), 18.42 (t, $CH_2CH_2CH_3$), 13.23 (t, $CH_2CH_2CH_3$) ppm; MS (TOF) *m/z*: 219.1 $[M + H]^+$; found 219.2.

(2*R*,3*R*,4*R*)-1-octyl-2-(hydroxymethyl)pyrrolidin-3,4-diol (20): To a solution of nitrone **30** (188 mg, 0.45 mmol) in MeOH (8 mL), Pd/C (420 mg) and concentrated HCl (four drops) were added. The mixture was stirred under H_2 atmosphere at rt for 15 h, then filtered through Celite and concentrated under reduced pressure to obtain the corresponding trihydroxy pyrrolidine chloridate (75 mg, 0.44 mmol) in 98% yield. This was dissolved in MeOH (3 mL), and then acetic acid (0.02 mL), $Na(CN)BH_3$ (28 mg, 0.44 mmol) and octanal (83 µL, 0.53 mmol) were added. The reaction mixture was stirred at rt for 8 h, then the solvent was removed under reduced pressure. The crude was passed through an ion-exchange resin Dowex WX8-200 and eluted with MeOH, water and a 6% solution of NH_4OH. Subsequent purification on silica gel (eluent: CH_2Cl_2/MeOH/NH_4OH 33% 10:1:0.1, R_f 0.1) gave pure **20** (37 mg, 0.15 mmol), in 33% yield as yellow pale oil. ^{1}H NMR (400 MHz, CD_3OD) δ 3.96**–**3.94 (m, 1H, H-4), 3.90**–**3.87 (m, 1H, H-3), 3.68 (m, 2H,

H-1'), 3.07 (d, J = 10.2 Hz, 1H, Ha-5), 2.88 (m, 1H, NCH_2), 2.73 (dd, J = 10.2, 4.4 Hz, 1H, Hb-5), 2.52 (bs, 1H, H-2), 2.43–2.37 (m, 1H, NCH_2), 1.54–1.50 (m, 2H), 1.31–1.30 (m, 10H), 0.89 (t, J = 6.8 Hz, 3H, *Me*) ppm; ^{13}C NMR (50.29 MHz, CD_3OD) δ 78.1 (d, C-3), 74.8 (d, C-4), 72.7 (d, C-2), 60.1 (t, C-1'), 58.3 (t, C-5), 54.8 (t, NCH_2), 30.7, 28.3, 28.1, 26.5, 26.2, 21.4 (t, CH_2), 12.1 (q, CH_3) ppm; ESIMS *m/z*: 246.33 ($[M + H]^+$, 100); Anal. calcd for $C_{13}H_{27}NO_3$: C, 63.64; H, 11.09; N, 5.71; found: C, 63.78; H, 11.15; N, 5.54.

(2*R*,3*R*,4*R*,5*R*)-3,4-bis(benzyloxy)-2-[(benzyloxy) methyl]-5-octylpyrrolidin-1-ol (31): To a solution of nitrone **30** (510 mg, 1.22 mmol) in dry THF (16 mL), a 2 M solution of octylmagnesium bromide in Et_2O (2.14 mmol) at −75 °C was added. After 3 h the temperature was raised to rt and the mixture controlled by TLC (eluent: petroleum ether/EtOAc 1:1) showing the disappearance of the starting material and the formation of a new product. The mixture was treated with a saturated aqueous solution of $NaHCO_3$ (12 mL) at 0 °C and extracted with Et_2O. The crude, obtained after evaporation of the solvent under reduced pressure, was purified by FCC (eluent: petroleum ether/EtOAc 5:1, R_f 0.2) to obtain pure **31** (540 mg, 1.02 mmol) in 84% yield as a white solid. ^{1}H NMR (400 MHz, $CDCl_3$) δ 7.32–7.25 (m, 15H, *Ar*), 4.55–4.42 (m, 6H, *Bn*), 3.93 (dd, J = 3.9, 2.9 Hz, 1H, H-3), 3.79 (dd, J = 5.1, 2.9 Hz, 1H, H-4), 3.77–3.75 (m, 1H, Ha-1'), 3.65–3.57 (m, 1H, Hb-1'), 3.52 (dd, J = 10.5, 5.1 Hz, 1H, H-2), 3.22–3.17 (m, 1H, H-5), 1.85–1.79 (m, 1H, CHCH_2), 1.58–1.53 (m, 1H), 1.50–1.43 (m, 1H, CHCH_2), 1.29–1.25 (m, 11H), 0.87 (t, J = 6.8 Hz, 3H, *Me*) ppm; ^{13}C NMR (50.29 MHz, $CDCl_3$) δ 138.2–138.1 (s, 3C, *Ar*), 128.5–127.5 (d, 15C, *Ar*), 86.8 (d, C-4), 84.6 (d, C-3), 73.4 (t, *Bn*), 71.7 (t, 2C, *Bn*), 70.1 (d, C-5), 70.0 (d, C-2), 68.3 (t, C-1'), 32.1 (t), 30.1–29.5 (t, 4C), 26.8 (t), 22.9 (t), 14.4 (q, CH_3) ppm; ESIMS *m/z*: 532.65 ($[M + H]^+$, 32), 554.75 ($[M + Na]^+$, 100); Anal. calcd for $C_{34}H_{45}NO_4$: C, 76.80; H, 8.53; N, 2.63; found: C, 76.53; H, 8.42; N, 2.36.

(2*R*,3*R*,4*R*,5*R*)-2-(hydroxymethyl)-5-octylpyrrolidin-3,4-diol (21): To a solution of hydroxypyrrolidine **31** (287 mg, 0.54 mmol) in MeOH (25 mL), Pd/C (150 mg) and HCl

37% (two drops) were added, and the mixture was stirred under H_2 atmosphere at rt overnight and then filtered through Celite and concentrated under reduced pressure. The obtained crude was passed through an ion-exchange resin Dowex WX8-200 eluting with MeOH, water and a 6% solution of NH_4OH, to obtain pure **21** (96 mg, 0.39 mmol) in 73% yield as a yellow solid, mp 113 °C (dec.). ^{1}H NMR (400 MHz, CD_3OD) δ 3.76 (t, *J* = 6.4 Hz, 1H, H-3), 3.67 (dd, *J* = 11.2, 3.9 Hz, 1H, Ha-1’), 3.62–3.56 (m, 2H, Hb-1’, H-4), 3.01 (dt, *J* = 6.3, 4.4 Hz, 1H, H-2), 3.89 (dt, *J* = 7.3, 5.4 Hz, 1H, H-5), 1.71–1.66 (m, 1H, CH_2), 1.47–1.41 (m, 1H, CH_2), 1.32–1.29 (m, 12H, CH_2), 0.89 (t, *J* = 6.8 Hz, 3H, *Me*) ppm; ^{13}C NMR (50.29 MHz, CD_3OD) δ 81.9 (d, C-4), 77.9 (d, C-3), 63.2 (d, C-2), 61.6 (t + d, 2C, C-5, C-1’), 33.7, 31.8, 29.6, 29.4, 26.5, 22.5 (t, 6C), 13.3 (q, CH_3) ppm; ESIMS *m*/*z*: 246.32 ($[M + H]^+$, 87), 268.46 ($[M + Na]^+$, 100); Anal. calcd for $C_{13}H_{27}NO_3$: C, 63.64; H, 11.09; N, 5.71; found: C, 63.69; H, 10.86; N, 5.49.
